# Time Window of the Critical Period for Neuroplasticity in S1, V1, and A1 Sensory Areas of Small Rodents: A Systematic Review

**DOI:** 10.3389/fnana.2022.763245

**Published:** 2022-03-17

**Authors:** Laís Resque Russo Pedrosa, Gabriele dos Santos Coimbra, Márcio Gonçalves Corrêa, Ivanira Amaral Dias, Carlomagno Pacheco Bahia

**Affiliations:** Laboratory of Neuroplasticity, Institute of Health Sciences, Federal University of Pará, Belém, Brazil

**Keywords:** critical period, neuroplasticity, primary sensory cortex, primary somatosensory cortex (S1), primary visual cortex (V1), primary auditory cortex (A1)

## Abstract

The plasticity of the central nervous system (CNS) allows the change of neuronal organization and function after environmental stimuli or adaptation after sensory deprivation. The so-called critical period (CP) for neuroplasticity is the time window when each sensory brain region is more sensitive to changes and adaptations. This time window is usually different for each primary sensory area: somatosensory (S1), visual (V1), and auditory (A1). Several intrinsic mechanisms are also involved in the start and end of the CP for neuroplasticity; however, which is its duration in S1, VI, and A1? This systematic review evaluated studies on the determination of these time windows in small rodents. The careful study selection and methodological quality assessment indicated that the CP for neuroplasticity is different among the sensory areas, and the brain maps are influenced by environmental stimuli. Moreover, there is an overlap between the time windows of some sensory areas. Finally, the time window duration of the CP for neuroplasticity is predominant in S1.

## Introduction

Neuroplasticity is the capacity of the central nervous system (CNS) to undergo structural and functional changes in response to environmental sensory experiences or even to adapt following injury (Ismail et al., 2017). Several genetic, molecular, and cellular mechanisms can modulate the synapses of neuronal circuits and cause functional improvement, loss, and/or behavioral changes (Johnston, 2009). The plasticity of the nervous tissue after stimuli or injury is more evident during the early postnatal period, i.e., a time window known as the critical period (CP) for neuroplasticity (Pascual-Leone et al., 2005). This short postnatal time window is characterized by heightened nervous system receptivity for adapting to stimuli provides a stable and long-term experiential foundation (Wiesel and Hubel, 1965).

Small rodents such as rats and mice have a lissencephalic brain with well-established primary sensory areas and have been used to investigate the CP for neuroplasticity (Stafford, 1984; Schlaggar et al., 1993; de Villers-Sidani et al., 2007). However, there is still no consensus on the time window start and end of all sensory areas. For instance, the CP for neuroplasticity of somatosensory (S1) may start at birth, i.e., P0 (Rice and Van Der Loos, 1977; Schlaggar et al., 1993), followed by visual (V1) and auditory (A1) areas (Stafford, 1984; Levine et al., 2017; Park et al., 2019).

Therefore, the CP for neuroplasticity can vary in accordance with the primary sensory areas related to different brain maps. In addition, environmental stimuli can influence the organization of S1, V1, and A1 in their respective sensorial maps. The effects of different stimuli and intrinsic mechanisms on the CNS have been investigated by several neuroscience researchers to establish the duration of the CP for neuroplasticity related to different primary sensory areas (Stafford, 1984; Goodman and Shatz, 1993; Schlaggar et al., 1993; Antonini et al., 1999; de Villers-Sidani et al., 2007, 2008; Fischer et al., 2007; Faguet et al., 2009; Zhou et al., 2011; Seelke et al., 2012; Levine et al., 2017; Park et al., 2019; van der Bourg et al., 2019). Considering the complexity of the CP for neuroplasticity and the respective time windows for each primary sensory area, preclinical studies can help to develop treatments for brain injuries such as the use of cochlear implants in cases of auditory deprivation (Kral, 2013). Therefore, this systematic review aimed to investigate and compare the time window of the CP for neuroplasticity in S1, V1, and A1 areas.

## Methods

### Protocol and Registration

This systematic review of animal studies was registered in a database (10.17605/OSF.IO/XNT6R) and conducted by following the Preferred Reporting Items for Systematic Reviews and Meta-Analyses (PRISMA) statement (Page et al., 2021).

### Eligibility Criteria, Information Sources, and Search

This Population/Problem, Interest, and Context (PICo) review included randomized controlled studies that evaluated neuroplasticity (P) through experimental interventions during the CP (I) and reported the time window duration (start and end) in the primary sensory areas of the cerebral cortex (Co).

English-language studies without the date of the publication restriction were searched in the PubMed, Web of Science, ScienceDirect, Embase, and Virtual Health Library databases by two reviewers (LP and GC) using the MeSH descriptors “neuronal plasticity,” “critical period,” and “cerebral cortex.” Additional primary studies were also searched throughout the reference lists of the retrieved articles. The search strategy was slightly adapted in each database and followed the PICo inclusion criteria described in the Supplementary Material. Search alerts for novel studies were also created.

### Study Selection and Data Collection

The studies were selected by following the criteria: (1) descriptor in the title or abstract, (2) English language, (3) original studies, and (4) animal model with small rodents. Reviews, case reports, descriptive studies, opinion articles, technical reports, guidelines, human, and *in vitro* studies were excluded.

The retrieved articles were uploaded to the Rayyan review assistance tool (https://rayyan.ai/) to exclude duplicated articles and studies with titles and abstracts that did not meet the eligibility criteria. After full-text reading, two independent reviewers (LP and GC) performed the final selection, and any disagreement was resolved by a third reviewer (CB). Then, the following data of the selected articles were extracted: publication year, analyzed cortical sensory area, characteristics of the animals, and main outcomes regarding the time window of the CP for neuroplasticity in S1, V1, and A1 areas.

### Risk of Bias in Individual Studies

The risk of bias for each study was independently assessed by two reviewers using the SYstematic Review Center for Laboratory animal Experimentation (SYRCLE), which is based on the Cochrane RoB tool and has been adjusted for biases that play a specific role in animal intervention studies (Hooijmans et al., 2014). The following domains were evaluated: allocation sequence, similarity among groups at baseline or confounder adjustment, randomized allocation of experimental and control groups, random housing conditions, blinding of caregivers and researchers, outcome assessor blinding, incomplete outcome data, selective outcome reporting, and other sources of bias. The interpretation of domains regarding allocation, housing, baseline similarities, and animal group assessment is known to influence the study outcomes. Hence, they were carefully analyzed to reduce the risk of bias and to guarantee the quality of this systematic review (van Zutphen et al., 2001).

In addition, “yes” or “no” questions were answered by the reviewers to assess the risk of bias: (1) Is there a possibility that the results are biased? (2) Do the results have factors that confuse the interpretation of the results? (3) Could the study results occur by chance? Articles that received mostly “no” answers were considered methodologically viable and with a low risk of bias.

## Results

### Study Selection

**Figure 1:**
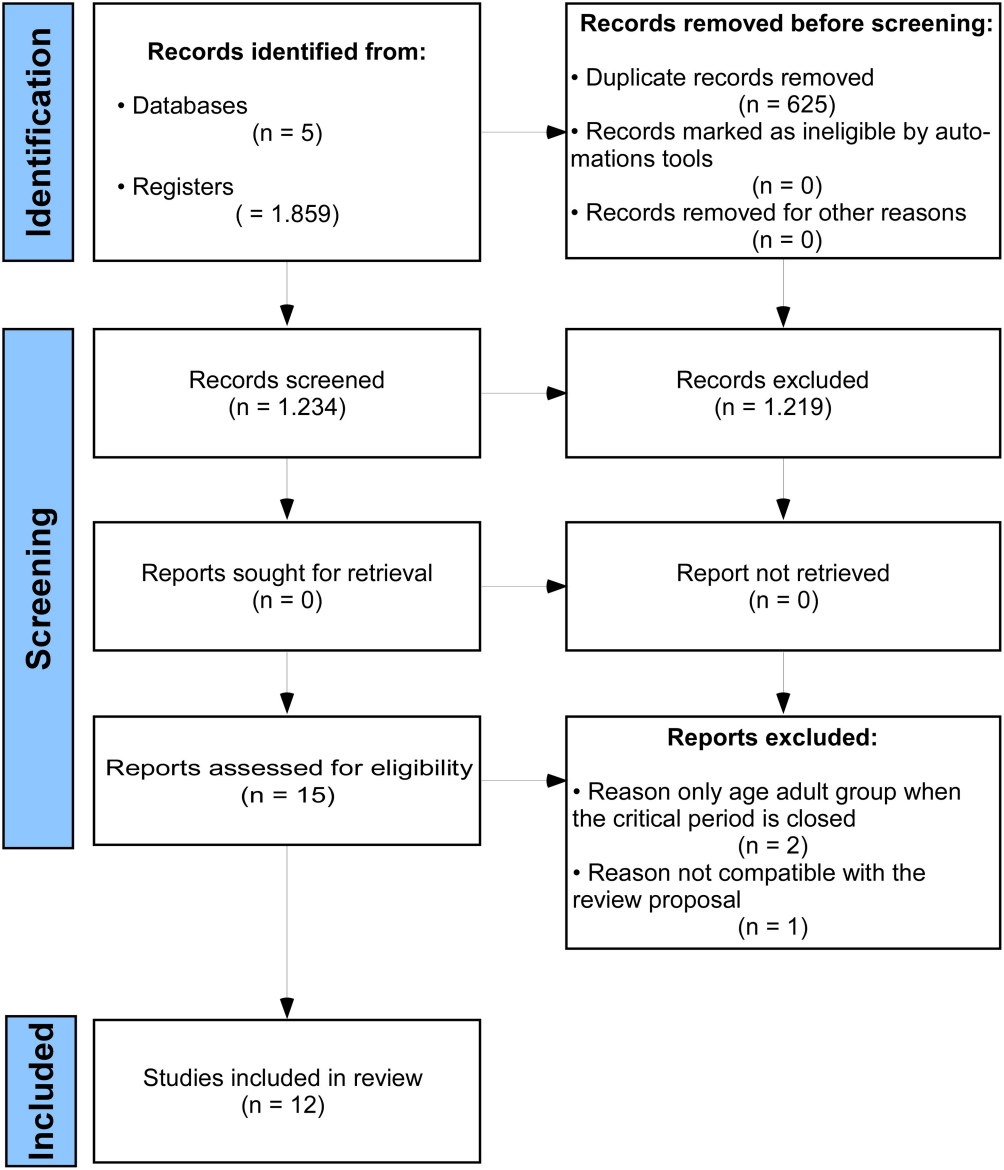
The Preferred Reporting Items for Systematic Reviews and Meta-Analyses (PRISMA) flow diagram.

Among 1,859 articles, 625 duplicates and other 1,219 articles were excluded during the title and abstract reading. Thus, 15 articles were fully read. Two articles were excluded since the CP for neuroplasticity was associated with neurodegenerative diseases, while one study did not meet the aim of this review. Then, 12 articles were eligible for the qualitative assessment (Figure 1).

### Study Characteristics

**Figure 2:**
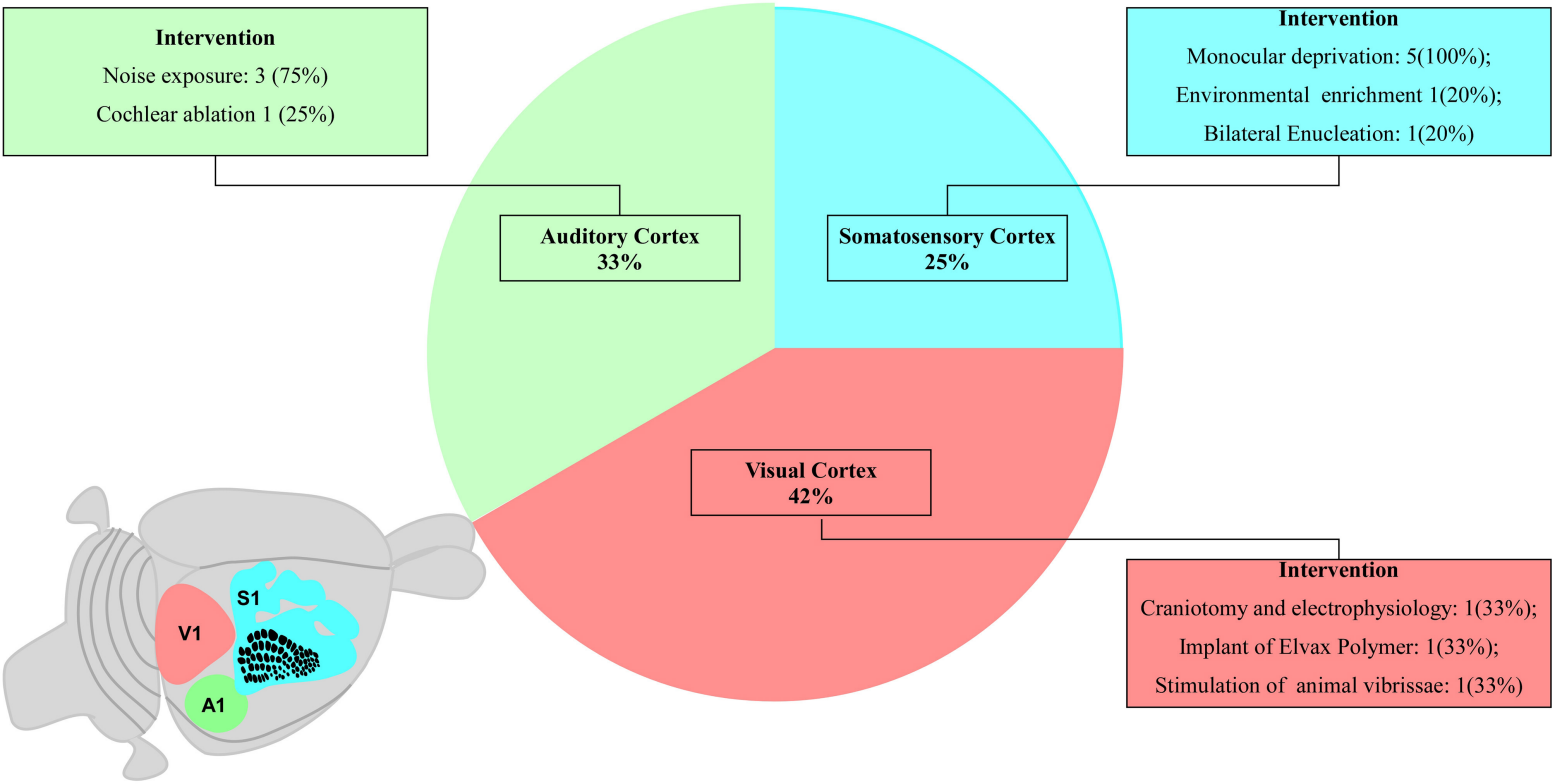
Schematic diagram proportionally shows the number of studies related to each primary sensory area. The schematic diagram of a small rodent brain highlights the primary sensory areas: three studies on the primary somatosensory cortex (S1, blue), four studies on the primary auditory cortex (A1, green), and five studies on the primary visual cortex (V1, pink).

Studies regarding the S1 area were performed by using electrophysiological recordings (Seelke et al., 2012), biopolymer implant loaded with glutamate receptor antagonist (Schlaggar et al., 1993), and standard stimulation patterns of the vibrissa system (van der Bourg et al., 2019). Researches on the V1 area reported some experimental interventions such as visual deprivation by eyelid suture (Stafford, 1984; Antonini et al., 1999; Fischer et al., 2007; Faguet et al., 2009; Levine et al., 2017) or monocular enucleation (Faguet et al., 2009), and environmental enrichment to increase the stimuli of V1 afferences (Levine et al., 2017). The investigation of the A1 area included continuous pure-tone exposure and electrophysiological recording of the auditory processing (de Villers-Sidani et al., 2007; Fischer et al., 2007), different standard sound stimuli (de Villers-Sidani et al., 2008), and/or cochlear ablation (Park et al., 2019). These characteristics are summarized in Figure 2 and fully described in the Supplementary Material.

The murine model was predominantly found in this review since seven studies used Sprague Dawley (Schlaggar et al., 1993; de Villers-Sidani et al., 2007, 2008; Zhou et al., 2011; Park et al., 2019) and Long Evans rat lineages (Stafford, 1984; Seelke et al., 2012) and five articles used C7BL6 mice (Antonini et al., 1999; Fischer et al., 2007; Faguet et al., 2009; Levine et al., 2017; van der Bourg et al., 2019).

### Risk of Bias Within Studies

**Figure 3:**
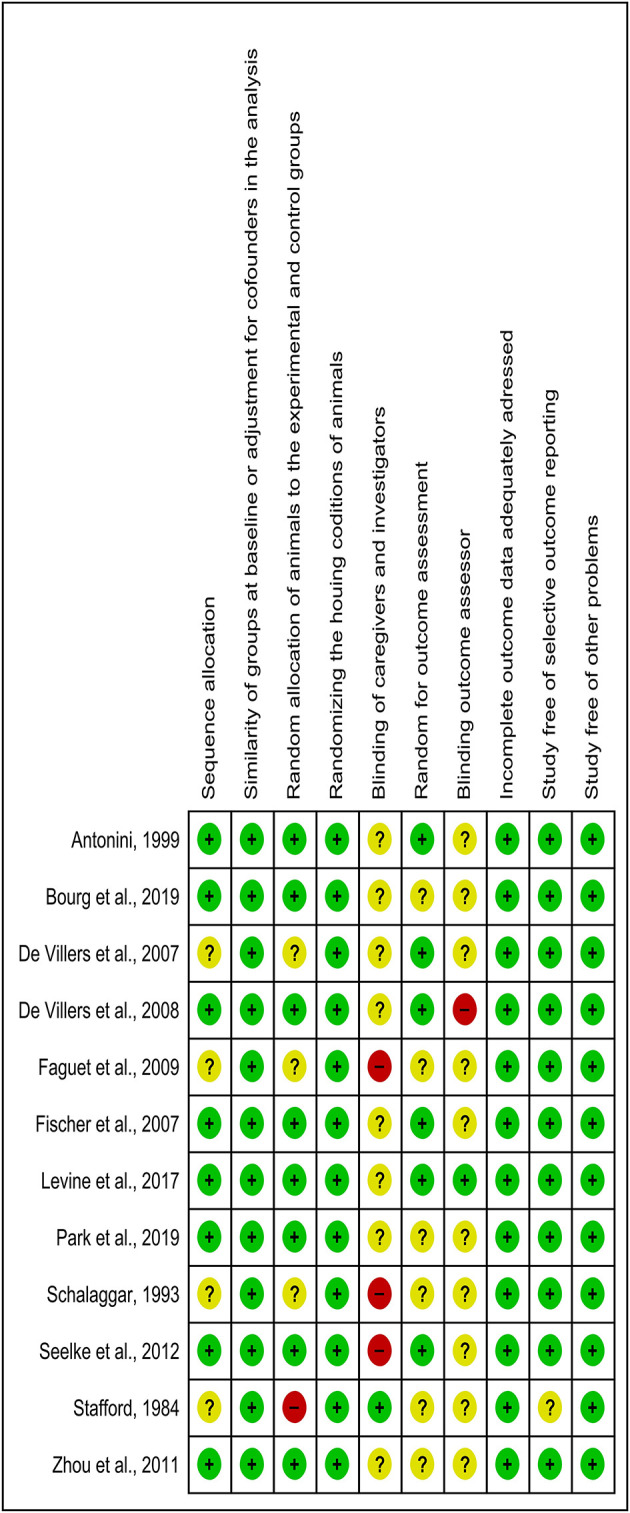
Risk of bias chart of studies included in the qualitative analysis by using 10 domains. Green, yellow, and red colors indicate a low, unclear, and high risk of bias, respectively.

**Figure 4:**
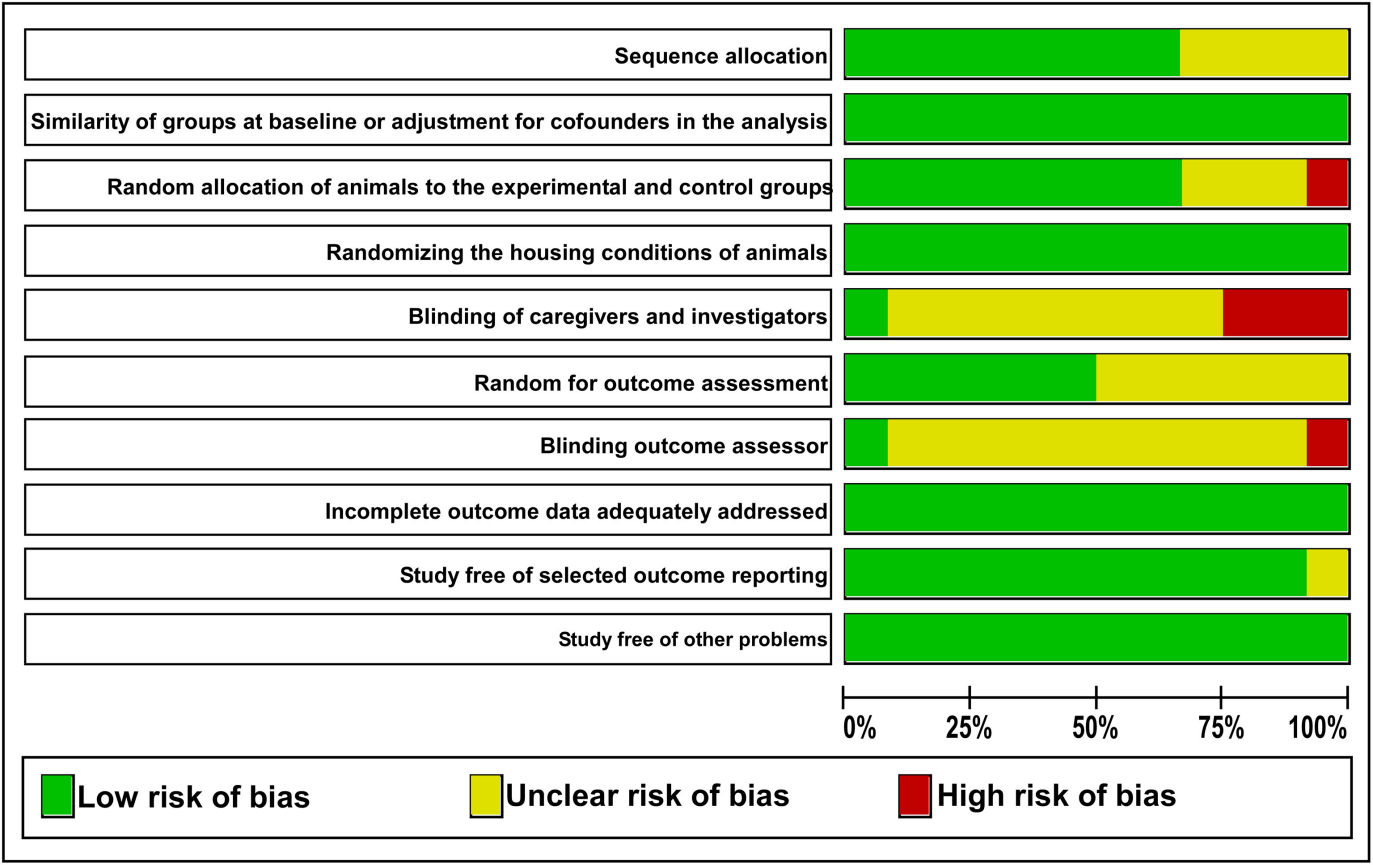
The judgments of the reviewer of each risk of bias item are presented as percentages. The green color indicates a low risk of bias. The yellow color indicates an unclear risk of bias. The red color indicates a high risk of bias.

The detailed analysis of the 10 main risk of bias domains indicated a low risk of bias regarding allocation sequence since most items were classified as “yes.” No study was classified with a high risk of bias. The domains “random housing conditions,” “incomplete outcome data,” and “others sources of bias” were found to be 100% adequate, while the “blinding of caregivers and researchers” and “outcome assessor blinding” were classified as high or unclear risk of bias in most of the studies (Figures 3, 4).

### Results of Individual Studies and Summary

The summary of the main results is shown in Table 1. Schlaggar et al. (1993) investigated the influence of postsynaptic activity on the time window of the CP for neuroplasticity in S1 during 5 days after birth. Other authors evaluated the S1 cortex development from birth until adulthood. Seelke et al. (2012) affirmed that postsynaptic inactivation interferes in the time window of the CP for neuroplasticity in the S1 area of rats submitted to vibrissae deprivation. Subsequently, all anatomical and functional alterations in S1 under sensorial deprivation from birth to early-adult lifetime were summarized on a topographic map. van der Bourg et al. (2019) evaluated the sharpening of response specificity to paired and sequential whisker deflections.

**Table 1:** Results of individual studies and synthesis of results.

**References**	**Cortical area**	**Objective**	**Experimental and control group**	**Main results**
Schlaggar et al. (1993)	S1	To test whether the somatosensory columns' developmental plasticity is similar to the competitive plasticity of ocular dominance columns, under postsynaptic blockade conditions	Sprague-Dawley rats at P0–P8 age; blockade of postsynaptic activation by inserting Elvax (polymer loaded with the glutamate receptor antagonist D-2-amino-5-phosphonovaleric acid for prolonged, controlled release) over the right S1 in P0 rat pups within 6 h of birth; electrocauterization of C row of vibrissae at either P0, P1, P2, P3, or P6; AChE activity analysis. Three groups: control group without subdural lesion, Control Elvax group loaded with a vehicle solution (inactive isomer of APV) and Elvax group	The interruption of postsynaptic activity reduced the responses to changes in the cortical region of both the compromised and adjacent barrel row. There is an important correlation between postsynaptic and presynaptic activity in the stabilization process of their connections and in the plasticity performance of the critical period
Seelke et al. (2012)	S1	To examine the developmental time course of the topography maps' emergence in S1 using rats as animal model	*Long Evans* rats at postnatal ages: P5, P10, P15, P20, and adults (P60); electrophysiological records from multiple sites in medial S1 correlating these data to architectonic distinctions; cytochrome oxidase; serotonin (5-HT) and myelin stainings	In P5, the S1 region is dominated by afferent vibrissae and its anatomical edges are already present. Topographic maps appear in the subsequent two weeks and become adult-like at the end of the third postnatal week (P20)
van der Bourg et al. (2019)	S1	To investigate the cortical processing of whisker-evoked responses and its maturation during development of mouse barrel cortex	C7BL/6 mice at P11–P27 age; single- and dual-whisker stimuli (C1 and C2) in a systematic manner; high-density multi electrode recordings *in vivo*; analyse de multi-unit activity and local potential fields across all layers of C1 and C2; cytochrome-oxidase (COX) histology to identify the barrel map	A gradual reduction in paired-pulse stimuli suppression during development related to development of early and distinct responses; the trans-columnar spread of early activity increased during development; Sequential activation of two neighborhood whiskers revealed a strong suppression of the second response, which was most pronounced in >P13 animals. Whisker stimulation evoked distinct responses and profiles in activated barrel columns due to significant changes in S1: greater temporal precision and sharpening of response specificity
Stafford (1984)	V1	To define the critical period in visually deprived mice	*Long Evans* rats by recording visual evoked potentials (VEPs) from the cortical surface after monocular deprivation at different stages of development between 2 and 10 weeks after birth	Monocular deprivation in young animals reduces both the visual acuity and the contrast sensitivity of the deprived eye and that the depth of attenuation depends upon the age at onset of deprivation. This suggests that the critical period is almost over by 6 weeks post-natally
Antonini et al. (1999)	V1	To elucidate the correlation between anatomical changes and functional plasticity in the development of the visual cortex, both during normal development and after plasticity induced by monocular deprivation	C57Bl/6 mice at P17–P60 age; monocular deprivation with eyelids trimmed within P17–P44; Tracer injections at lateral geniculate nucleus (LGN) sites to to reconstruct single axons serving the contralateral eye and innervating the binocular portion of V1; single-unit electrophysiological recordings; biocyting histochemistry; Phaseolus Vulgaris Leucoagglutitinin PHA-L immunohistochemistry	Monocular deprivation ending at P40 appeared to promote the growth of the open eye's contralateral projection without causing the closed eye's contralateral input to shrink. Continued deprivation to P60 prevented the growth of the closed eye's contralateral inputs
Fischer et al. (2007)	V1	To answer whether adult V1 can recover from prolonged DM	C57BL mice at P21 to P45 ages; long term monocular deprivation from early development to mature ages (well past the critical period); using electrophysiological methods	Partial recovery in V1 after deprivation and almost complete recovery of visual acuity and Ocular Dominance (OD) after reopening the eye followed by occlusion of the non-deprived eye. This findings suggest that adult visual experience can restore visual functions which fail to develop properly as a result of deprivation in early development
Faguet et al. (2009)	V1	To characterize possible differences in the contralateral and ipsilateral pathways at the peak of the critical plasticity period	C57BL mice at P-28 age, monocular deprivation by suture and contralateral eye enucleation; by imaging intrinsic optical responses	Visual deprivation results in a loss of the ability of cortical response to stimulation through the private eye. In addition, the ipsilateral eye pathway is affected by the quality of vision through the opposite eye. This findings indicate that although both contra and ipsilateral eye pathways require visual experience for their maintenance, ipsilateral eye projections bear an additional, unique sensitivity to binocular interactions
Levine et al. (2017)	V1	To determine the effect of Monocular Deprivation during the critical period and test whether Environmental Enrichment can rescue the post-critical period binocular correspondence	C57BL mice/6; monocular deprivation to P19/20 to P30/P40 ages; both single-unit electrophysiology and two-photon calcium imaging; environmental enrichment	The results show that for cells that are clearly dominated by one of the two eyes, the input representing the weaker eye changes its orientation preference to align with that of the dominant eye, to achieve binocularly matched orientation preference. These studies thus reveal ocular dominance as a key driver of the binocular matching process, consistent with a Hebbian mechanism whereby the dominant input instructs the weaker input to adopt its tuning properties
de Villers-Sidani et al. (2007)	A1	To examine the effects of exposure to pure tones on the auditory cortex of developing rats at different postnatal ages	*Sprague-Dawley* rats at P10 and P60 ages; stimulation of the left ear with 7 kHZ pure tones at eight sound intensities [0–70 dB] at a rate of two stimuli per second; electrophysiological recordings of a single or small cluster of A1 neurons; A1 cortical mapping according to eletrophyological characteristics	Evoked potentials at A1 are recorded for the first time at P10 and, by P14, all components of an adult-like evoked response (P60) are present Pure-tone exposure resulted in profound, persistent alterations in sound representations in A1 only if the exposure occurred during a brief period extending from postnatal day 11 (P11) to P13
de Villers-Sidani et al. (2008)	A1	To investigate whether the closing of the critical period should be considered as a uniform event or as a process controlled by progressive, local, activity-oriented changes in this cortical area	Sprague-Dawley rats before hearing onset at P7 and up to 1 week after the normal closure of the critical period for spectral tuning; different from auditory stimuli	The closing of the critical period is not unitary to the entire cortex, it occurs locally in cortical subregions according to experience, These results indicate that the control of the duration and closure of the critical period are dependent on the local state of cortical (or limited-sector system) maturation.
Zhou et al. (2011)	A1	Understanding the critical period should be seen as an early stage of development of brain growth	*Sprague Dawley* rats were exposed to moderate level continuous acoustic noise from Pw8–Pw13; auditory brainstem response measurement; quantitative immunoblotting and ELISA	There is a broad reversal of maturational changes that mark a substantial reversal of the adult functional state back toward a less mature. The present study indicates that this non-structured sensory bombardment can by itself drive a change in inhibitory and excitatory circuits, and a reduction in elements of the extracellular matrix linked to the reinstatement of plasticity in the cortex
Park et al. (2019)	A1	To examine the serial change of sound-specific auditory cortical activation patterns in age-matched normal hearing (NH) and young single-sided deafness (YSSD) rats to understand the critical period that influences a benefit of a binaural hearing	*Sprague-Dawley* rat at age; left-side cochlear ablations at the P10; sound stimulation of the right ear; multi neural recording in the bilateral auditory cortices during P14–P73; sound-evoked multi neural recording in bilateral auditory cortex.	NH group: larger peak amplitude and total responsive area of the contralateral hemisphere to sound stimulation in all ages. YSSD group: reactive area in the contralateral side was significantly smaller than that in the ipsilateral side at post-deafening (PD) 2 weeks (W) and PD4W, indicating the disappearance of contralateral dominance within PD4W. Monaural stimulation from the hearing ear exclusively activated the contralateral hemisphere at PD6W and PD8W that leads to loss of capacity for plastic reorganization. The early unilateral deafening leads to an alternation of contralateral dominance by a more rapid and massive reorganization toward the ipsilateral cortex.

Stafford (1984) originally investigated the time window of the CP for neuroplasticity in V1 at 2 (eye-opening), 4, 6, and 10 postnatal weeks by the modulation of the luminance stimulus. Conversely, Antonini et al. (1999) examined V1 responses from P17 to P60 and suggested that visual deprivation induces anatomical changes followed by functional plasticity until P30. In V1, the peak of the CP for neuroplasticity is influenced by the electrical activity of both contralateral and ipsilateral eye pathways (Faguet et al., 2009). However, visual deprivation associated with posterior environmental enrichment can induce the recovery of V1 plasticity after the post-CP P30/P40 (Fischer et al., 2007), albeit this late recovery was usually partial without significant changes in the neuronal network (Levine et al., 2017).

Three studies investigated the electrophysiological cortical responses in A1 at the second postnatal week (de Villers-Sidani et al., 2007, 2008; Park et al., 2019). The first potentials in A1 were recorded at P10 and P14, when the complete neural electrical response associated with the maturation of parvalbumin-positive inhibitory neurons were recorded (de Villers-Sidani et al., 2008). Stimuli from distinct harmonic sound patterns changed the time window of the CP for neuroplasticity in specific A1 subregions (de Villers-Sidani et al., 2008). Since early unilateral deafening reorganizes A1 ipsilateral, early intervention may be crucial to recovering binaural hearing (Park et al., 2019). Conversely, prolonged exposure to continuous noise after the end of the CP for neuroplasticity can cause a partial reversal of the maturation changes related to the adult functional state (Zhou et al., 2011).

## Discussion

**Figure 5:**
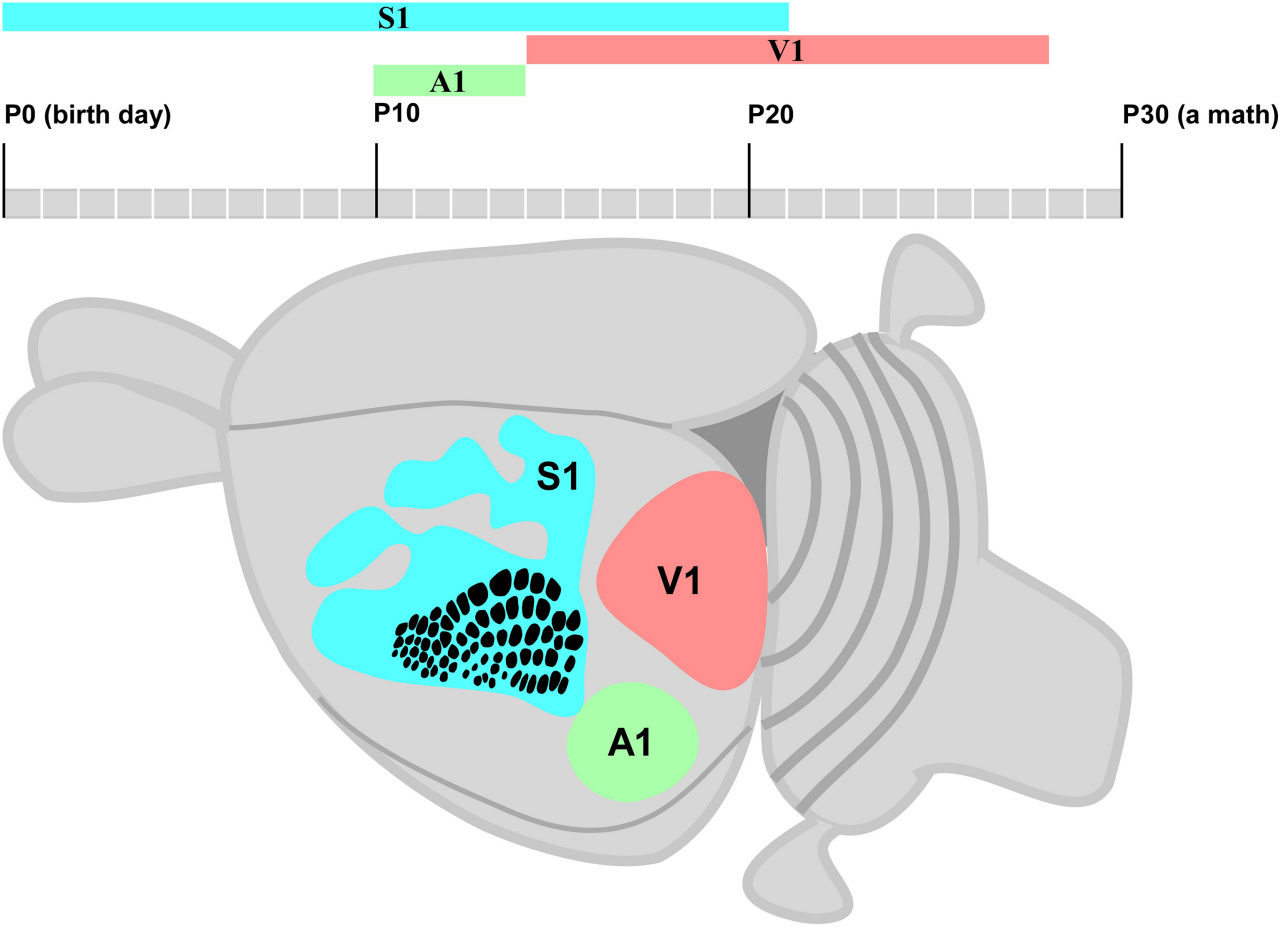
Schematic diagram of a small rodent brain highlights S1 (blue), A1 (green), and V1 areas (pink). The posteromedial barrel subfield (PMBSF) cortex in S1 is highlighted by a point-to-point representation of whiskers; rows and receptive fields are represented by a single whisker.

This systematic review aimed to assess the time windows of the CP for neuroplasticity in small rodents. The findings of selected studies indicated different time windows for each primary sensory area (S1, V1, and A1). The time window of the CP for neuroplasticity in the S1 area starts early, followed by A1 and V1 areas. S1 and A1 were found with the longest and shortest time windows, respectively. Moreover, the S1 completely and partially overlaps the A1 and V1 time windows of the CP, respectively (Figure 5).

Considering that the outcome of a systematic review depends on the methodological quality of the studies (Hooijmans et al., 2014), 10 domains were used to avoid a high risk of bias, and any essential domain was considered with a high risk of bias (Figures 3, 4). Both “blinding of caregivers and researchers” and “outcome assessor blinding” were considered “unclear” in most studies since some conditions or outcome assessments were not accurately detailed. However, these domains were not considered essential for assessing the methodological quality.

Although small rodents are widely used to investigate neuroplasticity, only 12 studies met the eligibility criteria (Figure 1). These animals are easy to handle, and their mechanisms of molecular glues or cellular events involved in the CP for neuroplasticity are very similar to those observed in large mammals (Reagan-Shaw et al., 2008). Thus, anatomical, physiological, and biochemical mechanisms investigated in experimental models with rats and mice have provided applicable findings regarding the time window of the CP for neuroplasticity (Reagan-Shaw et al., 2008).

The time window of the CP for neuroplasticity usually starts at neurogenesis followed by neuronal migration during the first postnatal week (Berry and Rogers, 1965). The time window in S1 starts earlier than in the V1 and A1 areas (Figure 5). The fundamental mechanisms of the brain formation of mice and rats are under continued debate. Thus, the early cortical patterning centers such as fibroblast growth factors (FGFs) have been suggested to define the border of each cortical area by controlling the expression of regulatory genes (Schubert et al., 2015). These genes encode cell adhesion molecules such as cadherins and axon guidance molecules such as ephrins, which characterize neocortex arealization (O'Leary and Wilkinson, 1999; Pallas, 2001). The shift of the gamma-aminobutyric acid (GABA) from excitatory to inhibitory activity is also an important subject (Represa and Ben-Ari, 2005). Furthermore, the end of the time window of the CP for neuroplasticity is modulated by GABAergic interneurons that restrict the pruning of excess connections: Hebbian Theory (Martens et al., 2015). In addition, environmental stimuli play an important role to connect the thalamocortical afferents to the correct cortical primary sensory area (Hanganu-Opatz, 2010). Interestingly, the peak of the time window of the CP for neuroplasticity may occur halfway of topographic map formation (Seelke et al., 2012) and at different moments from the first postnatal day in the S1, V1, and A1 areas (Figure 5).

This systematic review indicated that the time window of the CP for neuroplasticity in S1 starts at P0 (Figure 5) due to the influence of intrinsic and extrinsic factors. For instance, sensory deprivation at the early postnatal development of whiskers changes the functional and morphological organization of the barrel field (Schlaggar et al., 1993) and leads to adult-like anatomical and functional S1 map (Seelke et al., 2012). All findings related to the time window of the CP for neuroplasticity in S1 were found from P0 to P21. The endocannabinoid CB1 receptor and brain-derived neurotrophic factor (BDNF) may regulate the development of interneurons in a specific S1 subregion such as the barrel field, forelimb, hind limb, and trunk (Li et al., 2009; Seelke et al., 2012; van der Bourg et al., 2019).

The time window of the CP for neuroplasticity can simultaneously occur in different cortical areas. The CP for neuroplasticity in A1 only starts when the time window in S1 is already halfway (Figure 5), followed by the refinement of cerebral cortical responses and simultaneous significant changes in the cochlea (Kalish et al., 2020). Since the duration of the CP for neuroplasticity in A1 may not extend beyond the first month of postnatal life (Park et al., 2019), early auditory stimulus seems important for the development of binaural hearing (Polley et al., 2013). It is very complex to determine the time window of the CP for neuroplasticity in A1 since different sound levels stimulate only some specific subregions (de Villers-Sidani et al., 2008), and the inhibitory neuronal activity is involved in the bilateral hearing development and the maturation of auditory refinement responses that end within P11–P14 (Kalish et al., 2020). Similar to S1, the extension of the CP for neuroplasticity in A1 may occur due to environmental stimuli that modulate intrinsic mechanisms such as continuous BDNF downregulation and tropism change of GABA receptor subunits (Zhou et al., 2011; refer to Figure 5).

The time window of the CP for neuroplasticity ends in A1 when it virtually starts in V1 represented by the eyes of the small rodent opening at P14. Stafford (1984) reported that monocular deprivation causes severe effects from the second up to the fourth postnatal week. However, the reorganization of contralateral and ipsilateral eye pathways is less affected at P40 (Antonini et al., 1999). Similar to S1 and A1, the time window of the CP for neuroplasticity in V1 is very complex to understand since the regulatory molecules involved in the determination of binocular zone are very different from those related to the monocular zone (Fischer et al., 2007; Faguet et al., 2009; Levine et al., 2017). At the same time, the environmental visual stimulus also activates the expression of some immediate early genes (IEGs) related to neuroplasticity modulation (Mower et al., 2002; Taha et al., 2002).

The data available in the studies of this systematic review indicate that the time window of the CP for neuroplasticity (Table 1) remains unclear (Ribot et al., 2021) since this complex event involves several different knowledge subjects (Wang et al., 2010; Erzurumlu and Gaspar, 2012; Levelt and Hübener, 2012; Takesian et al., 2018). In addition, it involves different molecular, cellular, and morphological mechanisms in S1, A1, and V1 that are influenced by environmental stimuli. This background supports novel approaches for further clinical trials on the treatment of reversible sensory deprivations by using prostheses, implants, and robotic exoskeletons.

## Conclusion

Although both the start and end of the time window of the CP for neuroplasticity in S1, V1, and A1 are still not precisely determined, this review roughly estimated the time window in the primary sensory areas of small rodents in the function of several intrinsic and extrinsic factors. In addition, S1 showed the longest time window of the CP for neuroplasticity followed by V1 and then A1. Due to its complexity and variability, the determination of both the start and end of the CP for neuroplasticity and the mechanisms involved is still unclear. Moreover, advanced clinical trials on therapies for neuronal dysfunctions are encouraged to better understand its clinical relevance for humans.

## Data Availability Statement

The original contributions presented in the study are included in the article/Supplementary Material, further inquiries can be directed to the corresponding author/s.

## Author Contributions

CB contributed to the study concept and design, obtained funding, and performed supervision. LP, GC, and MC contributed to data acquisition. LP and GC contributed to the drafting of the manuscript. ID and CB contributed to critical revision for important intellectual content. All authors had full access to all data and took responsibility for the integrity of the information, text, and the accuracy of the analysis. All authors contributed to the article and approved the submitted version.

## Funding

This review was financially supported by the National Council for Scientific and Technological Development (CNPq, grants 310054/2018-4, 447835/2014-9, 483404/2013-6, 444967/2020-6, and 444982/2020-5) and the Coordination for the Improvement of Higher Education Personnel (CAPES, grant PROCAD 21/2018).

## Conflict of Interest

The authors declare that the research was conducted in the absence of any commercial or financial relationships that could be construed as a potential conflict of interest.

## Publisher's Note

All claims expressed in this article are solely those of the authors and do not necessarily represent those of their affiliated organizations, or those of the publisher, the editors and the reviewers. Any product that may be evaluated in this article, or claim that may be made by its manufacturer, is not guaranteed or endorsed by the publisher.
