# Supplementary material for: Time Window of the Critical Period for Neuroplasticity in S1, V1, and A1 Sensory Areas of Small Rodents: A Systematic Review
Source: Front Neuroanat. 2022 Mar 17;16:763245. doi: 10.3389/fnana.2022.763245 (PMC8970055; doi:10.3389/fnana.2022.763245)
Supplement: Supplementary file 1 [file Data_Sheet_1.docx]

Title: Time window of the Critical Period of neural plasticity in small rodent’s sensory cortices S1, V1, and A1: a systematic review

1. PICOS Search Strategy

PICo: “How long does the critical period of neuronal plasticity last in the cerebral cortex?”

|  | **Key-words** | **MESH** | **ENTRY TERMS** |
| --- | --- | --- | --- |
| **P** | Neuronal plasticity | Neuronal plasticity | Neuronal Plasticity  Neuronal Plasticities  Plasticities, Neuronal  Synaptic Plasticity  Plasticities, Synaptic  Plasticity, Synaptic  Synaptic Plasticities  Neuroplasticity  Neuroplasticities  Neural Plasticity  Neural Plasticities  Plasticities, Neural  Plasticity, Neural  Plasticity, Neuronal  Brain Plasticity  Brain Plasticities  Plasticities, Brain  Plasticity, Brain |
| **I** | Critical Period | Critical Period | ---------------------- |
| **Co** | Cerebral Cortex | Cerebral Cortex | Cerebral Cortex  Cerebral Cortex  Cortex, Cerebral  Cortices, Cerebral  Cerebrus, Cortex  Cortex Cerebrus |

| **Database** | **Total articles** |
| --- | --- |
| PubMed | 823 |
| Web of Science | 218 |
| Science Direct | 182 |
| Embase | 10 |
| Virtual Health Library | 626 |
| Total | 1.859 |
| **Duplicates removed** | 625 |
| **Included articles** | 12 |

Search strategy in PubMed

(("neuronal plasticity"[MeSH Terms] OR ("neuronal"[All Fields] AND "plasticity"[All Fields]) OR "neuronal plasticity"[All Fields] OR ("neuronal plasticity"[MeSH Terms] OR ("neuronal"[All Fields] AND "plasticity"[All Fields]) OR "neuronal plasticity"[All Fields] OR ("neuronal"[All Fields] AND "plasticities"[All Fields]) OR "neuronal plasticities"[All Fields]) OR ("neuronal plasticity"[MeSH Terms] OR ("neuronal"[All Fields] AND "plasticity"[All Fields]) OR "neuronal plasticity"[All Fields] OR ("plasticities"[All Fields] AND "neuronal"[All Fields])) OR ("neuronal plasticity"[MeSH Terms] OR ("neuronal"[All Fields] AND "plasticity"[All Fields]) OR "neuronal plasticity"[All Fields] OR ("synaptic"[All Fields] AND "plasticity"[All Fields]) OR "synaptic plasticity"[All Fields]) OR ("neuronal plasticity"[MeSH Terms] OR ("neuronal"[All Fields] AND "plasticity"[All Fields]) OR "neuronal plasticity"[All Fields] OR ("plasticities"[All Fields] AND "synaptic"[All Fields]) OR "plasticities synaptic"[All Fields]) OR ("neuronal plasticity"[MeSH Terms] OR ("neuronal"[All Fields] AND "plasticity"[All Fields]) OR "neuronal plasticity"[All Fields] OR ("plasticity"[All Fields] AND "synaptic"[All Fields]) OR "plasticity synaptic"[All Fields]) OR ("neuronal plasticity"[MeSH Terms] OR ("neuronal"[All Fields] AND "plasticity"[All Fields]) OR "neuronal plasticity"[All Fields] OR ("synaptic"[All Fields] AND "plasticities"[All Fields]) OR "synaptic plasticities"[All Fields]) OR ("neuronal plasticity"[MeSH Terms] OR ("neuronal"[All Fields] AND "plasticity"[All Fields]) OR "neuronal plasticity"[All Fields] OR "neuroplasticity"[All Fields] OR "neuroplastic"[All Fields]) OR ("neuronal plasticity"[MeSH Terms] OR ("neuronal"[All Fields] AND "plasticity"[All Fields]) OR "neuronal plasticity"[All Fields] OR "neuroplasticities"[All Fields]) OR ("neuronal plasticity"[MeSH Terms] OR ("neuronal"[All Fields] AND "plasticity"[All Fields]) OR "neuronal plasticity"[All Fields] OR ("neural"[All Fields] AND "plasticity"[All Fields]) OR "neural plasticity"[All Fields]) OR ("neuronal plasticity"[MeSH Terms] OR ("neuronal"[All Fields] AND "plasticity"[All Fields]) OR "neuronal plasticity"[All Fields] OR ("neural"[All Fields] AND "plasticities"[All Fields]) OR "neural plasticities"[All Fields]) OR ("neuronal plasticity"[MeSH Terms] OR ("neuronal"[All Fields] AND "plasticity"[All Fields]) OR "neuronal plasticity"[All Fields] OR ("plasticities"[All Fields] AND "neural"[All Fields])) OR ("neuronal plasticity"[MeSH Terms] OR ("neuronal"[All Fields] AND "plasticity"[All Fields]) OR "neuronal plasticity"[All Fields] OR ("plasticity"[All Fields] AND "neural"[All Fields]) OR "plasticity, neural"[All Fields]) OR ("neuronal plasticity"[MeSH Terms] OR ("neuronal"[All Fields] AND "plasticity"[All Fields]) OR "neuronal plasticity"[All Fields] OR ("plasticity"[All Fields] AND "neuronal"[All Fields]) OR "plasticity neuronal"[All Fields]) OR ("neuronal plasticity"[MeSH Terms] OR ("neuronal"[All Fields] AND "plasticity"[All Fields]) OR "neuronal plasticity"[All Fields] OR ("brain"[All Fields] AND "plasticity"[All Fields]) OR "brain plasticity"[All Fields]) OR ("neuronal plasticity"[MeSH Terms] OR ("neuronal"[All Fields] AND "plasticity"[All Fields]) OR "neuronal plasticity"[All Fields] OR ("brain"[All Fields] AND "plasticities"[All Fields]) OR "brain plasticities"[All Fields]) OR ("neuronal plasticity"[MeSH Terms] OR ("neuronal"[All Fields] AND "plasticity"[All Fields]) OR "neuronal plasticity"[All Fields] OR ("plasticities"[All Fields] AND "brain"[All Fields])) OR ("neuronal plasticity"[MeSH Terms] OR ("neuronal"[All Fields] AND "plasticity"[All Fields]) OR "neuronal plasticity"[All Fields] OR ("plasticity"[All Fields] AND "brain"[All Fields]) OR "plasticity brain"[All Fields])) AND ("critical period, psychological"[MeSH Terms] OR ("critical"[All Fields] AND "period"[All Fields] AND "psychological"[All Fields]) OR "psychological critical period"[All Fields] OR ("critical"[All Fields] AND "period"[All Fields]) OR "critical period"[All Fields]) AND ("cerebral cortex"[MeSH Terms] OR ("cerebral"[All Fields] AND "cortex"[All Fields]) OR "cerebral cortex"[All Fields] OR ("cerebral cortex"[MeSH Terms] OR ("cerebral"[All Fields] AND "cortex"[All Fields]) OR "cerebral cortex"[All Fields]) OR ("cerebral cortex"[MeSH Terms] OR ("cerebral"[All Fields] AND "cortex"[All Fields]) OR "cerebral cortex"[All Fields] OR ("cortex"[All Fields] AND "cerebral"[All Fields]) OR "cortex cerebral"[All Fields]) OR ("cerebral cortex"[MeSH Terms] OR ("cerebral"[All Fields] AND "cortex"[All Fields]) OR "cerebral cortex"[All Fields] OR ("cortices"[All Fields] AND "cerebral"[All Fields]) OR "cortices cerebral"[All Fields]) OR ("cerebral cortex"[MeSH Terms] OR ("cerebral"[All Fields] AND "cortex"[All Fields]) OR "cerebral cortex"[All Fields] OR ("cerebrus"[All Fields] AND "cortex"[All Fields])) OR ("cerebral cortex"[MeSH Terms] OR ("cerebral"[All Fields] AND "cortex"[All Fields]) OR "cerebral cortex"[All Fields] OR ("cortex"[All Fields] AND "cerebrus"[All Fields])))) AND (fha[Filter])

Search strategy in Web of Science

((TS=( (Neuronal Plasticity) OR(Neuronal Plasticities) OR (Plasticities, Neuronal) OR (Synaptic Plasticity) OR (Plasticities, Synaptic) OR (Plasticity, Synaptic) OR (Synaptic Plasticities) OR (Neuroplasticity) OR (Neuroplasticities) OR (Neural Plasticity) OR (Neural Plasticities) OR (Plasticities, Neural) OR (Plasticity, Neural) OR (Plasticity, Neuronal) OR (Brain Plasticity) OR (Brain Plasticities) OR (Plasticities, Brain) OR (Plasticity, Brain))) AND TS=((Critical Period))) AND TS=((Cerebral Cortex) OR (Cerebral Cortex) OR (Cortex, Cerebral) OR (Cortices, Cerebral) OR (Cerebrus, Cortex) OR (Cortex Cerebrus)) Filter by: Language: English

Search strategy in Science Direct

"Neuronal Plasticity" AND "Critical Period" AND "Cerebral Cortex"

Search strategy in Embase

(neuronal AND plasticity OR (neuronal AND plasticities) OR (plasticities, AND neuronal) OR (synaptic AND plasticity) OR (plasticities, AND synaptic) OR (plasticity, AND synaptic) OR (synaptic AND plasticities) OR neuroplasticity OR neuroplasticities OR (neural AND plasticity) OR (neural AND plasticities) OR (plasticities, AND neural) OR (plasticity, AND neural) OR (plasticity, AND neuronal) OR (brain AND plasticity) OR (brain AND plasticities) OR (plasticities, AND brain) OR (plasticity, AND brain)) AND critical AND period AND (cerebral AND cortex OR (cortex, AND cerebral) OR (cortices, AND cerebral) OR (cerebrus, AND cortex) OR (cortex AND cerebrus))

Search strategy in Virtual Health Library

((Neuronal Plasticity) OR(Neuronal Plasticities) OR (Plasticities, Neuronal) OR (Synaptic Plasticity) OR (Plasticities, Synaptic) OR (Plasticity, Synaptic) OR (Synaptic Plasticities) OR (Neuroplasticity) OR (Neuroplasticities) OR (Neural Plasticity) OR (Neural Plasticities) OR (Plasticities, Neural) OR (Plasticity, Neural) OR (Plasticity, Neuronal) OR (Brain Plasticity) OR (Brain Plasticities) OR (Plasticities, Brain) OR (Plasticity, Brain)) AND ((Critical Period)) AND ((Cerebral Cortex) OR (Cerebral Cortex) OR (Cortex, Cerebral) OR (Cortices, Cerebral) OR (Cerebrus, Cortex) OR (Cortex Cerebrus))
